# Supplementary material for: Explicit Tracking of Uncertainty Increases the Power of Quantitative Rule-of-Thumb Reasoning in Cell Biology
Source: Biophys J. 2014 Dec 2;107(11):2612–7. doi: 10.1016/j.bpj.2014.08.040 (PMC4255194; doi:10.1016/j.bpj.2014.08.040)
Supplement: Document S1. Additional supplemental information [file mmc1.pdf]

Explicit tracking of uncertainty increases the power of quantitative rule-of-thumb reasoning in cell biology – Supplementary Information

Iain G. Johnston, Benjamin C. Rickett, and Nick S. Jones

Technical Details

This text describes various technical details of the web interface for Caladis, our probabilistic calculator, at `www.caladis.org`.

Distributions and sampling. Available distributions in Caladis are normal, uniform, discrete uniform, log-normal, binomial, Poisson, beta, exponential, gamma, and geometric. For each distribution identified in the input expression, Caladis uses Monte Carlo sampling to sample the resultant distribution: each iteration, random samples are drawn from each characterised input distribution and the value of the input expression is calculated and recorded to build up the resultant distribution. The user may determine the number of iterations to employ. Resultant distributions for which the summary statistics have not converged are identified and a warning message encouraging the use of robust statistics (median, IQR) or more samples is displayed.

Options. Caladis users can select the number of Monte Carlo samples, the angle unit (degrees or radians) and binning methods (Freedman-Diaconis, Scott, or Sturges approaches). Additionally, users can elect whether to perform standard deviation analysis, and how various values from the Bionumbers repository are interpreted (see below).

Bionumber selection. Bionumber IDs may be directly entered (e.g. `#100001`), or found with a built-in browser (Fig. 2C). This browser enables a user to identify a Bionumber for use in calculations using one of three approaches. Firstly, a given Bionumber may be selected directly from a full listing of all available experimental data. Secondly, a user may navigate through the set of organisms for which Bionumbers are available, and through the types of value present for each organism (classified by the units with which Bionumbers are associated, so that, for example, length scales may be distinguished from reaction rates). Thirdly, a user may search the descriptions of all Bionumbers for terms of interest, then select from the available search results. Upon identification of a Bionumber of interest, the user may automatically enter that Bionumber (with its associated experimental uncertainty) into their calculation. Upon entry, a Bionumber's distribution will automatically be assigned to the input expression (see below).

Bionumber distributions. Caladis assigns distributions to Bionumbers by the format of their associated range information, according to a protocol dictated by the user. Data presented as '$x \,\text{to}\, y$', '$x - y$', or similar, may be interpreted as $U(x, y)$, a uniform distribution between x and y; or $\exp(N(m, s))$, a log-normal distribution with parameters chosen such that x and y are $\pm 1\sigma$ points of the distribution. This log-normal interpretation is accomplished through the mapping $m = (\ln a + \ln b)/2; s = m - \ln a$, so that the mean and standard deviation of the resultant log-normal distribution are $\mu = \exp(m + s^2/2)$ and $\sigma = \sqrt{(\exp(s^2) - 1)\exp(2m + s^2)}$. Data presented as '$x \pm y$' may be interpreted as either normal or log-normal with mean x and standard deviation y. Hence, for example, Bionumber #100001 (the cell length of *E. coli*) is listed as '1.94 to 2.72 μm', so we may interpret it (in addition to its qualitative details) as $U(1.94, 2.72)$ in units of μm. In the case of Bionumbers with no associated information regarding uncertainty, Caladis automatically assigns a normal distribution with a coefficient of variation of 0.5 (which the user can change manually).

Documentation. The online documentation at `www.caladis.org/tutorial` contains extensive information on the available mathematical functions and operators, syntax, and details of the optional choices. Several example calculations, illustrating syntax and descriptions of probability distributions, are available on the Caladis input screen, along with options regarding the mathematical details of the calculation.
